# Mixed sorbent in miniaturized stir bar sorptive dispersive microextraction for the determination of gut microbiome metabolites in plasma samples

**DOI:** 10.1007/s00216-026-06391-8

**Published:** 2026-02-19

**Authors:** Cristian Azorín, Sara R. Fernandes, Luisa Barreiros, Juan L. Benedé, Alberto Chisvert, Marcela A. Segundo

**Affiliations:** 1 GICAPC Research Group, Department of Analytical Chemistry, University of Valencia, 46100 Burjassot, Valencia Spain; 2 LAQV, REQUIMTE, Department of Chemical Sciences, Faculty of Pharmacy, University of Porto, Rua de Jorge Viterbo Ferreira 228, 4050-313 Porto, Portugal; 3 ESS, Polytechnic of Porto, Rua Dr. António Bernardino de Almeida 400, 4200-072 Porto, Portugal

**Keywords:** Low-volume samples, Gut microbiome metabolites, Miniaturized stir bar sorptive dispersive microextraction, Plasma, Hydrophilic interaction liquid chromatography

## Abstract

An analytical method for the simultaneous determination of three gut microbiome metabolites related to the gut-brain axis (trimethylamine *N*-oxide (TMAO), phenylacetylglutamine (PAG), and 4-ethylphenyl sulfate (EPS)) in human plasma is presented. The proposed method is based on miniaturized stir bar sorptive dispersive microextraction (mSBSDME) followed by liquid chromatography-tandem mass spectrometry (LC-MS/MS). In this work, a magnetic composite made of CoFe_2_O_4_ magnetic nanoparticles embedded into a mixture of commercial sorbents (hydrophilic-lipophilic balance and mixed-mode cation exchange) was employed as magnetic sorbent material, taking advantage of its affinity to the target analytes. Under the optimized conditions, the method was validated and showed good analytical features in terms of linearity (at least up to 1000 ng mL^−1^), limits of detection (22, 2, and 9 ng mL^−1^ for TMAO, PAG, and EPS, respectively), repeatability (RSD ≤ 10%), and accuracy (91–101%). Moreover, relative recoveries between 84 and 104% were obtained, showing matrix effects were negligible using deuterated standards as surrogates. This new approach was successfully applied to clinical samples, allowing the rapid determination of the target metabolites in a single run for the first time.

**Supplementary Information:**

The online version contains supplementary material available at 10.1007/s00216-026-06391-8.

## Introduction

The gut-brain axis is a complex bidirectional communication system that connects the gastrointestinal tract and the brain. This intricate network involves the central nervous system, the enteric nervous system, and the gut microbiota [1]. Emerging research highlights its pivotal role in regulating various aspects of physical and mental health. The gut-brain axis influences not only digestion but also humor, cognition, and even immune function [2]. Signals travel along this axis through neurotransmitters, hormones, and immune molecules, allowing the gut to convey information to the brain and vice versa [3]. Gut microbiota affects hosts and other bacteria by producing several kinds of metabolites such as short-chain fatty acids, bile acids, and choline metabolites, among others. These metabolites can induce a series of physiological and pathological functions on hosts and other bacteria, such as regulation of the composition and function of gut microbiota, influence on nutrition absorption and the intestinal barrier and gut motility, modulation of host metabolism and circadian rhythm, and impact on the systemic immune response, the nervous system and drug efficacy and toxicity [4]. In this sense, gut microbiota has been linked to several illnesses in recent years, including autism, anxiety, obesity, schizophrenia, Parkinson’s disease, and Alzheimer’s disease, underscoring the profound impact of gut health on overall well-being [5].

Among the vast variety of gut microbiota metabolites, those which have been directly related to diseases are of great interest for their potential use as prognostic biomarkers. For example, trimethylamine *N*-oxide (TMAO) is produced when trimethylamine, a waste product of gut microbes, is converted via hepatic flavin monooxygenases [6]. Elevated circulating levels of TMAO increase the risk of cardiovascular diseases and have been related to diseases such as ischemic stroke, arrhythmia, and diabetic complications [6–9]. Plasma metabolome study also demonstrated the existence of gut microbiota-derived uremic toxins [10]. Among them, phenylacetylglutamine (PAG) and 4-ethylphenyl sulfate (EPS) have been related to cardiovascular and neurodegenerative diseases [11–13] and autism spectrum disorder [14, 15], respectively. Therefore, the determination of these metabolites is of great interest, and they constitute the target analytes of this work, whose relevant information can be found in Table 1.

**Table 1:**
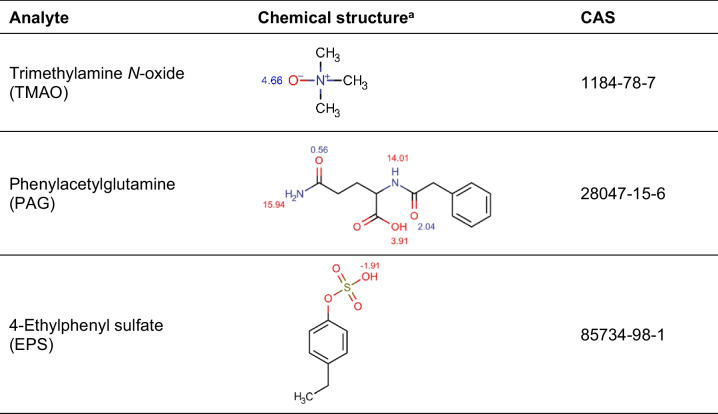
Chemical structure, calculated pK_a_, and CAS number of the target compounds

^a^Structures show next to each functional group its calculated pK_a_. Calculations were performed using Chemicalize software (https://chemicalize.com)

It should be emphasized that bioanalysis often entails the detection of substances in complex biological matrices at extremely low concentrations. Hence, selective and sensitive methods are necessary, typically combining (micro)extraction techniques with highly sensitive detectors. In this sense, chromatographic systems (both liquid (LC) and gas chromatography (GC)) coupled to mass spectrometry (MS) have been extensively used for the determination of TMAO [16–31], PAG [19, 32–37] and EPS [22, 37, 38] in different biological matrices, such as urine, plasma/serum, saliva, and feces. However, to the best of our knowledge, they have not been jointly determined. The expected levels for these compounds vary in a wide range depending on the condition of the subject under study, being approximately 40–5600 ng mL^−1^ for TMAO [39], 130–870 ng mL^−1^ for PAG [40] and 25–4800 ng mL^−1^ for EPS [38].

Among the extensive collection of microextraction techniques, dispersive-based techniques present considerable relevance in the sample preparation field because the high contact area between the sorbent and the sample solution favors the mass transfer, thus improving kinetics and reducing extraction times [41]. Besides, the use of magnetic (nano)materials (i.e., magnetic nanoparticles (MNPs) or composite materials combining MNPs and polymers) permits their easy retrieval by applying an external magnetic field [42, 43]. In this context, a hybrid dispersive-based microextraction approach that uses magnetic materials as extraction phase, termed stir bar sorptive dispersive microextraction, was developed [44] and recently miniaturized for application to low-volume samples [45]. This new methodology allows a rapid determination of the target analytes employing a few microliters of sample and organic solvents, and sorbent amounts in the mg level. This permits the analysis of barely available samples and fits the principles of Green Sample Preparation [46]. Moreover, the extraction assembly described for this technique allows to extract up to 15 samples simultaneously for high-throughput sample preparation [45]. More recently, the sample throughput of this methodology was expanded by its adaptation to the 96-well format [47].

The aim of this work is to present a high-throughput method based on miniaturized stir bar sorptive dispersive microextraction (mSBSDME) for the preparation of small volumes of plasma samples prior to LC-MS/MS analysis targeting the determination of three gut microbiome metabolites, namely TMAO, PAG, and EPS. As a magnetic sorbent, cobalt ferrite MNPs embedded into a mixture of polymers were employed. Different commercial sorbents and combinations were evaluated to obtain the most appropriate and effective composite.

## Experimental

### Reagents

Trimethylamine *N*-oxide dihydrate ≥ 99.0% and phenylacetyl-L-glutamine ≥ 95.0% were acquired from Sigma-Aldrich (St. Louis, MO, USA), and 4-ethylphenyl sulfate potassium salt 96% was provided by TRC Canada (North York, ON, Canada). Trimethylamine-d_9_ *N*-oxide 98%, phenylacetyl-d_5_-L-glutamine 98%, and 4-ethylphenyl-d_4_ sulfate potassium salt 95% used as surrogate standards were also provided by TRC Canada.

For the preparation of synthetic plasma as a surrogate matrix, as described in Supplementary Information, sodium chloride 100%, magnesium sulfate 99%, and formic acid ≥ 99% from VWR Chemicals (Leuven, Belgium); potassium chloride 99.5–100.5% from Fluka (Seelze, Germany); sodium hydrogen carbonate 99.9% from Fisher Chemical (Loughborough, UK); calcium chloride 99.0–107.0%, disodium hydrogen phosphate dihydrate 98.0–101.0%, and bovine serum albumin (BSA) ≥ 98% from Sigma-Aldrich; and sodium dihydrogen phosphate monohydrate 99.02–102.2% from Merck (Darmstadt, Germany) were used.

Ultrapure water was obtained from an Easy 15 water purification system provided by Heal Force (Shanghai, China).

LC-MS grade acetonitrile from Merck, ammonium formate ≥ 99% from Sigma-Aldrich, and formic acid ≥ 99% from VWR Chemicals were used to prepare the mobile phase.

Nitrogen used as nebulizer and drying gas in the MS/MS ion source was obtained by a nitrogen generator Genius 1050 from Peak Scientific (Inchinnan, UK). Extra pure Argon BIP® (99.999%), used as collision gas in the MS/MS collision cell, was provided by Air Products (Allentown, PA, USA).

For the synthesis of CoFe_2_O_4_ MNPs, cobalt(II) chloride hexahydrate 99.9% and iron(III) chloride hexahydrate 99.0% were purchased from Acros Organics (Fair Lawn, NJ, USA), and sodium hydroxide (reagent grade) ≥ 98.5% was purchased from Scharlau (Barcelona, Spain).

Five commercial sorbents with 60 μm particle size (Oasis® HLB, Oasis® MCX, Oasis® MAX, Oasis® WCX, and Oasis® WAX) were purchased from Waters Corp. (Milford, MA, USA). The sorbents were obtained in cartridge format, dismantled, and the sorbent beads were saved in a clean glass container. Ethanol absolute used for the magnetization of sorbents was supplied by VWR.

Methanol for HPLC gradient-grade from VWR was employed for standard solution preparation and in the extraction.

### Instrumentation

For the LC-MS analysis, a Nexera X2 UHPLC chromatographic system comprising two LC-30AD pumps, a DGU-20A5R degassing unit, a SIL-30AC autosampler, and a CTO-20AC oven from Shimadzu Corporation (Kyoto, Japan) was employed. The MS/MS system was a triple quadrupole LCMS-8040 mass spectrometer equipped with an electrospray ionization source (ESI) from Shimadzu Corporation.

For the synthesis of the magnetic nanoparticles, a heating plate with magnetic stirrer from Stuart Scientific (Staffordshire, UK) and an oven from J.P. Selecta (Barcelona, Spain) were used. For the magnetization of sorbents, a 10-position stirring plate RO 10 from IKA (Staufen, Germany) and an oven Incuterm I-40 from Raypa (Terrassa, Spain) were employed.

The mSBSDME procedure was conducted using a custom-designed multiextraction platform comprising a MX-3K magnetic stirrer (18 W, 0–3000 rpm) from Anzeser (Frankfurt am Main, Germany), integrated with a 3D-printed support specifically designed to accommodate 15 flat-base glass inserts. Comprehensive information regarding the design and manufacturing of this extraction platform is available in the original work where the workstation was first introduced [45]. Miniaturized extraction vessels consisted of 400-μL glass inserts (31 mm in height and 4 mm inner diameter) from Labbox (Barcelona, Spain). To enable dispersion and subsequent collection of the magnetic sorbent within the donor phase, cylindrical neodymium magnets (3 mm length × 2 mm diameter, 45 MGO) were employed, supplied by Supermagnete (Gottmadingen, Germany).

### Synthesis of magnetic composite

The synthesis of the magnetic composite consisted of two steps: (1) the synthesis of the cobalt ferrite magnetic nanoparticles (CoFe_2_O_4_ MNPs) by wet chemical co-precipitation according to an adapted protocol [48], and (2) subsequent embedding of MNPs on the sorbent. Both steps were described elsewhere [49]. Some slight modifications were made for the second one, though.

In short, 0.15 g of the MNPs and 0.15 g of sorbent beads and 50 mL of ethanol were stirred at room temperature for 24 h. Finally, the solid was magnetically decanted and washed with ethanol to eliminate the non-magnetic sorbent and then filtered under vacuum through an 11-µm pore size filter paper in order to discard the free MNPs and isolate the composite. It was dried in an oven at 70 °C overnight and finally grinded into a fine powder.

### Preparation of standard and sample solutions

Individual standard solutions were prepared at 1.0 mg mL^−1^ in water (TMAO, EPS) or ethanol (PAG) and were kept at −20 °C protected from light to avoid possible degradation. From these solutions, an intermediate multicomponent solution of 10 μg mL^−1^ in water was prepared and kept in the same conditions. This solution was diluted daily to 5 μg mL^−1^ in water. From this solution, working standard solutions for calibration (10–1000 ng mL^−1^) in surrogate matrix (synthetic plasma, prepared as described in Supplementary Information) were prepared for subsequent extraction.

Similarly, individual surrogate standard solutions were prepared at 1.0 mg mL^−1^ in methanol and were kept at −20 °C protected from light. From these solutions, an intermediate multicomponent solution of 10 μg mL^−1^ in water was prepared and kept in the same conditions. This solution was diluted daily to 3 μg mL^−1^ in the same solvent.

Blood was collected by trained nurses from eight adults participating in the Microbi-A study. The study complied with the 1964 Helsinki Declaration and all subsequent revisions and followed the generally accepted norms of good clinical practices. Furthermore, the study protocol for Microbi-A was approved by the Ethics Committee for Health of *Faculdade de Psicologia e de Ciências da Educação da Universidade do Porto* (approval number 2020/12-01b). The signed informed consent of each participant was previously obtained. Immediately after collection, blood samples were centrifuged at 5000 rpm (3892 × g) for 15 min at 4 °C for plasma separation. Plasma was frozen and stored at −80 °C. Before analysis, plasma samples were thawed, vortexed, and used directly.

In order to get the solutions for the mSBSDME procedure, both working standard solutions and plasma samples were prepared as follows: 60 µL of plasma or standard solution was vortex-mixed with 10 µL of surrogates standard solution (3 μg mL^−1^) and 230 µL of formic acid (0.75 M).

### mSBSDME procedure

**Fig. 1:**
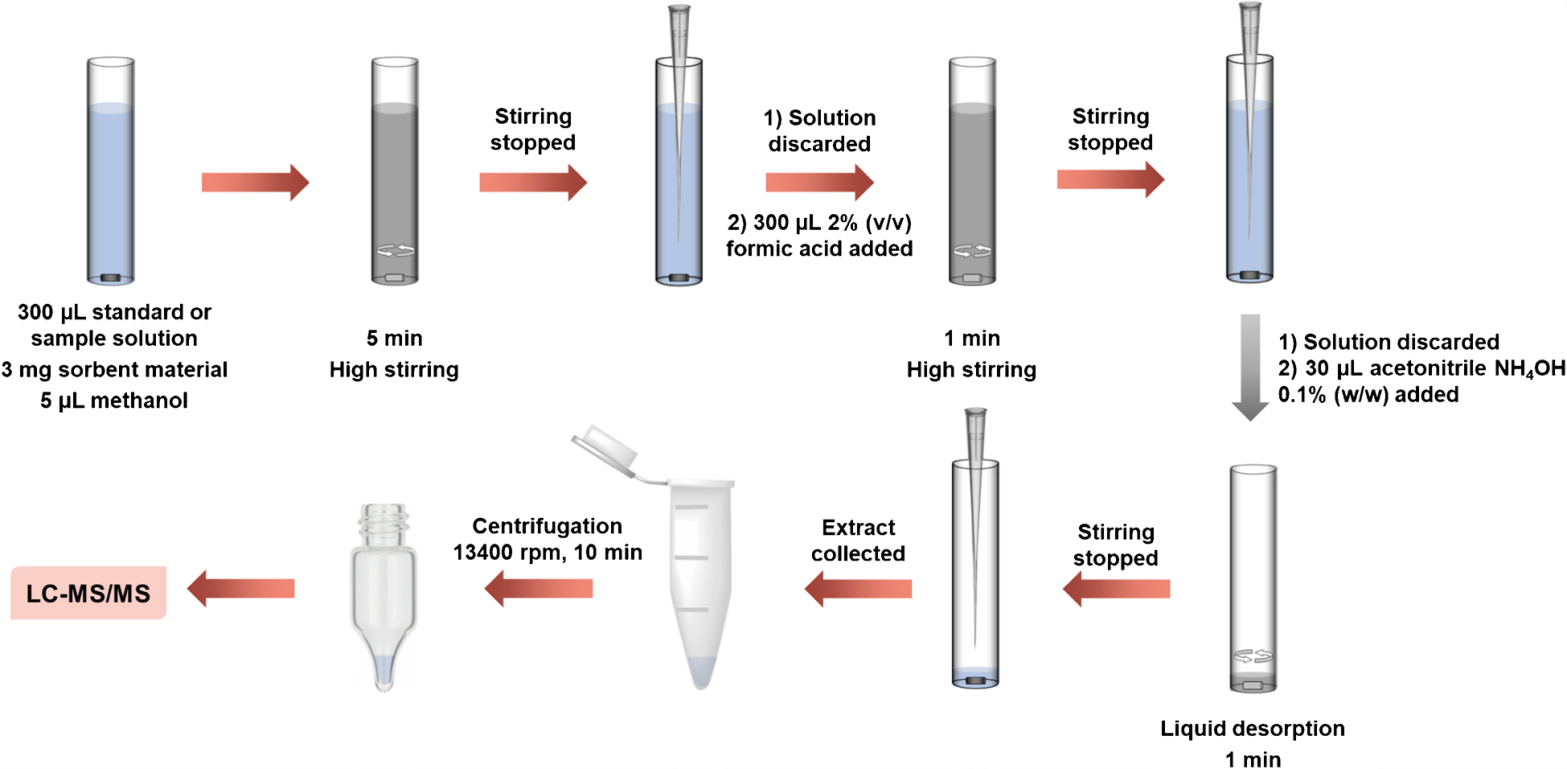
Schematic diagram of the proposed mSBSDME-LC-MS/MS method

Three milligrams of the magnetic composite was weighed into flat-based glass inserts and a neodymium magnet (2 mm diameter; 3 mm length) was introduced in each insert. Subsequently, 5 µL of methanol was added to assist dispersion of the material and to reduce the surface tension of water, facilitating the introduction of the 300 µL of previously prepared standard or sample solutions. Then, they were vigorously stirred for 5 min to achieve total dispersion of the composite within the solutions, thus favoring the extraction of the analytes. Afterwards, the stirring was stopped, and the inserts were left still so that the magnetic composite was retrieved by the magnetic field and recoated the stir bar again. Next, the solutions were taken carefully with a gel loading pipette tip and discarded. Then, the inserts, magnets, and composites were washed with 300 µL of an aqueous solution of formic acid 2% (v/v) and stirred for 1 min. The washing solutions were also discarded. In order to carry out the liquid desorption of the analytes, 30 µL of acetonitrile containing 0.1% (w/w) of NH_4_OH was introduced into the inserts. After 1 min of vigorous stirring, the extracts were transferred to centrifuge microtubes and centrifuged at 12,100 g (13,400 rpm) for 10 min to ensure any particle was settled and prevent it from entering the chromatographic system. After extraction and elution, the magnetic sorbent coating the stir bar was not recovered for reuse, as its cleaning would require significant solvent consumption to avoid carryover, resulting in a greater environmental impact than disposal. Finally, the supernatants were transferred to conical-base injection vials and subjected to LC-MS/MS analysis. Figure 1 shows a schematic diagram of the proposed method.

### LC-MS/MS analysis

Separations were carried out in a hydrophilic interaction chromatography (HILIC) LUNA® diol-based silica column (150 × 2 mm, 3 µm particle size) from Phenomenex (Torrance, CA, USA). One microliter of each solution was injected into the chromatographic system. The mobile phase consisted of solvent A (water containing ammonium formate 10 mM, pH 3.2) and solvent B (acetonitrile-water (90:10, v/v) containing ammonium formate 10 mM, pH 3.2), at a mixing ratio of 5:95, in such a way that there was a proportion of acetonitrile of ca. 85% (v/v) in the final mobile phase composition, also ensuring buffer solubility. Isocratic elution was implemented at a flow rate of 0.3 mL min^−1^, and the column temperature was kept constant at 40 °C. The run time was 4 min.

The triple quadrupole MS detector was operated in electrospray ionization mode (ESI), by multiple reaction monitoring (MRM). MS operating parameters were defined as follows: nebulizing gas (nitrogen, 2.6 L min^−1^), drying gas (nitrogen, 15 L min^−1^), heat block temperature (425 °C), desolvation line temperature (300 °C), detector voltage (2.02 kV), and collision gas (argon, 230 kPa). The *m/z* values of precursor and product ions, and the corresponding collision energy values are shown in Table S1. The ions to be monitored were selected taking into account not only high signal intensities but also the consistency with the chemical structure of the target analytes. Annotated product ion spectra can be found in Supplementary Information (Figs. S1–S3). TMAO and PAG were analyzed in positive ionization mode, whereas EPS was determined in negative ionization mode. All selected ion transitions were recorded during the entire chromatographic run. The LabSolutions software version 5.60 SP2 from Shimadzu Corporation (Kyoto, Japan) was applied for peak detection and quantification.

### Method validation

The quality parameters of the proposed method, such as linearity, extraction efficiency (EE), limits of detection (LOD) and quantification (LOQ), accuracy, and precision, were defined and evaluated according to the concepts and terminology described in the ICH Q2(R2) harmonized guideline on validation of analytical procedures [50].

The linearity was studied by measuring standard solutions at different concentrations from 10 to 1000 ng mL^−1^ prepared in surrogate matrix, all of them containing 500 ng mL^−1^ of the surrogate standards. Each standard was subjected to the proposed method and injected three times. Calibration curves were plotted using the ratio of the peak area of each target analyte to its surrogate standard (A_i_/A_S_) versus the analyte concentration in ng mL^−1^.

The slope of two calibration curves (100–1000 ng mL^−1^), one of them prepared in synthetic plasma and subjected to the proposed method and the other one prepared in the desorption solvent and directly injected into the LC-MS/MS without any extraction, was compared. EEs were calculated considering the ratio of the slopes compared to the maximum theoretical enrichment factor.

The LODs and LOQs were calculated as 3 and 10 times, respectively, the standard deviation of different blank solutions subjected to the proposed method.

The accuracy and precision of the method were evaluated applying the proposed method to quality control (QC) standard solutions in surrogate matrix containing the target analytes at three levels of concentration (i.e., 30, 400 and 800 ng mL^−1^) and 500 ng mL^−1^ of the surrogates.

## Results and discussion

### Selection of chromatographic conditions

Given the polar nature of the target compounds (Table 1), HILIC mode was chosen for chromatographic separation. The use of a buffered mobile phase and the presence of a counter ion are usually essential to achieve improved peak shape and retention in HILIC due to ionic interactions between the liquid layer on the stationary phase surface and the charged analytes [51]. Therefore, ammonium formate was selected as the buffer system instead of formic acid, exploiting its volatility for MS. However, its limited solubility in acetonitrile must be considered. A maximum of 90% (v/v) of acetonitrile can be present in the mobile phase components to prevent buffer precipitation. Hence, final mobile phase mixtures were achieved by mixing solvent A (water containing ammonium formate) and solvent B (acetonitrile-water (90:10, v/v) containing ammonium formate). The effect of chromatographic conditions, namely mobile phase parameters such as pH, proportion of acetonitrile, flow rate, and buffer concentration, on the separation of target analytes was evaluated. The selection was based on the retention times of the three analytes that allowed achieving a resolution factor ≥ 1.5 in the shortest time.

The retention in chromatography is clearly influenced by the charge state of the analytes as a result of the pH of the media. What may seem a trivial reasoning looking at the pH of mobile phase and the pK_a_(s) of the analytes, can get very tricky when analytes possess multiple ionizable moieties, as is the case with the three target compounds, leading to complex species distributions (Figs. S4–S6). It is also important to consider the high organic content of the mobile phase (ca. 90%) where the actual pH is 1–1.5 pH units closer to neutral than in aqueous media [52]. In this sense, different pHs of mobile phase were tested (pH 3.2, 6.6 and 8.0). For this purpose, a 100 mM aqueous solution of ammonium formate was prepared and adjusted to the desired pH with formic acid or ammonium hydroxide, as needed. Then it was diluted 20 times with water and acetonitrile for mobile phase components A and B, respectively. As starting point, 90% (v/v) of acetonitrile in the final mobile phase composition and a concentration of 5 mM of ammonium formate were set for the study of the effect of pH on separation. As shown in Table 2 (Set A), while EPS was not influenced by pH because it is negatively charged in the whole pH range (Fig. S6), TMAO and PAG were highly retained at higher pH due to their ionized species (Fig. S4 and S5), leading to longer runs and wider peaks. Therefore, pH 3.2 was selected for further studies.

Once pH was selected, different amounts of acetonitrile in the final mobile phase composition were tested (Set B). As expected in HILIC mode, lower amounts of acetonitrile increased elution strength and decreased retention times. An intermediate proportion of 80% (v/v) was selected to ensure suitable resolution of peaks in the minimum time.

**Table 2:** Sets of experiments for chromatographic conditions selection

Set	% (v/v) Acetonitrile	Buffer concentration (mM)	pH	Flow rate (mL min^−1^)	Retention time (min)			Resolution factor^a^	
					EPS	PAG	TMAO	EPS-PAG	PAG-TMAO
A	90	5	3.2	0.3	1.57	3.57	5.42	10.1	3.0
			6.6		1.42	4.05	11.20	12.9	5.8
			8.0		1.53	11.52	> 20	35.8	> 6.4
B	90	5	3.2	0.3	1.57	3.57	5.42	10.1	3.0
	80				1.40	1.78	2.33	2.1	2.1
	70				1.43	1.64	1.96	1.1	1.6
C	80	5	3.2	0.3	1.40	1.78	2.33	2.1	2.1
				0.2	2.10	2.69	3.52	2.6	2.2
				0.1	4.24	5.29	6.76	2.7	2.1
D	80	5	3.2	0.2	2.10	2.60	3.24	2.1	2.1
		10			2.15	2.63	3.05	2.1	1.5
		15			2.16	2.62	2.99	2.0	1.3
		20			2.17	2.64	2.96	2.0	1.3
E	90	10	3.2	0.3	1.52	3.24	3.62	10.4	1.0
	85				1.45	2.13	2.60	3.6	1.7
	80				1.42	1.79	2.13	2.0	1.4

^a^Calculated as 2(t_R,2_−t_R,1_)/(W_1_ + W_2_), where t_R,i_ is the retention time of the compound and W_i_ is the peak width at the base

Then, different flow rates were studied (Set C), in an attempt to separate the first peak (i.e., EPS) from the solvent front, but results were similar, and therefore, the highest flow rate (0.3 mL min^−1^) was selected to get shorter runs and faster analysis.

Different buffer concentrations were subsequently tested to study its effect on analyte retention and peak shape (Set D). At higher concentrations, TMAO was less retained, and the peak shape (i.e., tailing) was considerably improved. The higher concentration that did not compromise the resolution between PAG and TMAO (resolution factor ≥ 1.5) was selected, i.e., 10 mM. Finally, considering the selected buffer concentration, the amount of organic modifier was revisited (Set E), selecting a final percentage of 85%, at which resolution factors were higher than 1.5.

**Fig. 2:**
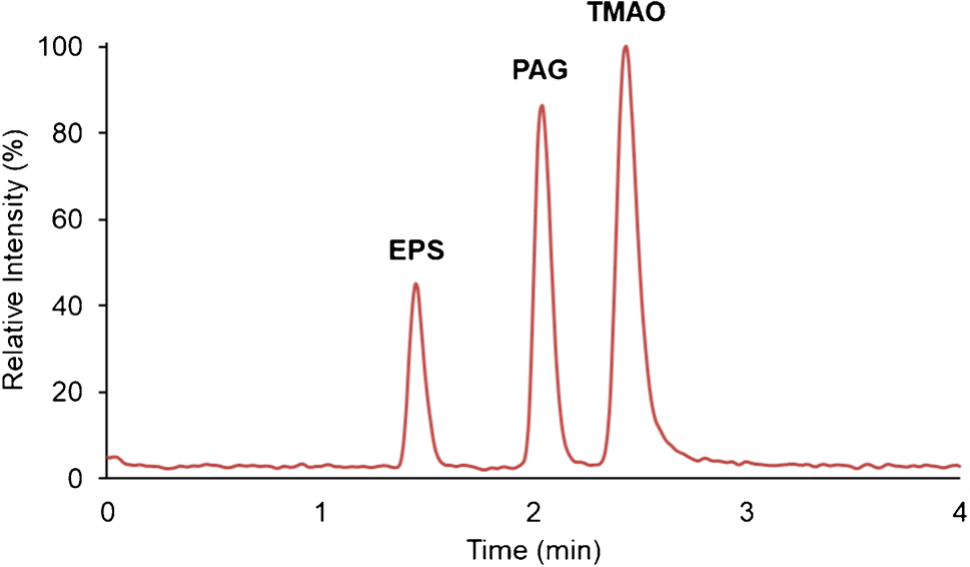
Representative chromatogram of a 600-ng mL^−1^ mixed standard solution obtained under the selected conditions (mobile phase: acetonitrile-water (85:15, v/v) containing ammonium formate 10 mM, pH 3.2, at a flow rate of 0.3 mL min^−1^). The represented signal is the sum of the three quantitative transitions (*m/z* 200.9>121.2 for EPS, *m/z* 265.2>130.1 for PAG, *m/z* 76.2>58.2 for TMAO)

Figure 2 shows a chromatogram of a standard solution obtained under the finally selected conditions.

### Selection and characterization of the sorbent material

Sorbent selection when analytes are so chemically different and also possess multiple ionizable moieties is usually a challenge. In this work a whole variety of commercial sorbents were considered, including hydrophilic-lipophilic-balanced polymers (Oasis® HLB), weak ion exchange (Oasis® WCX and WAX), and mixed-mode strong ion exchange sorbents (Oasis® MCX and MAX).

**Fig. 3:**
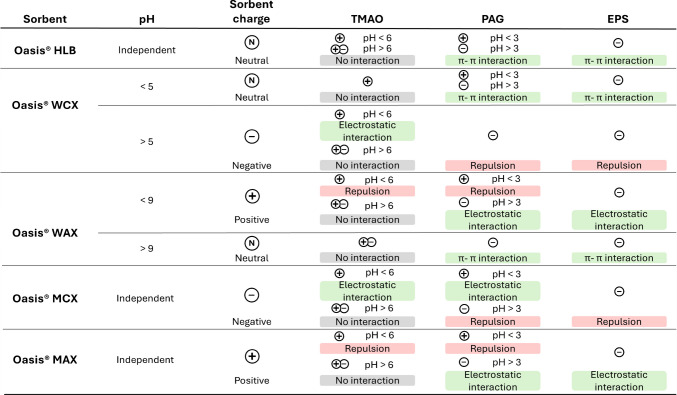
Summary of interactions between analytes and sorbents at different pH values

First, the possible interactions between each sorbent and analyte were theoretically studied at different pH ranges taking into account the microspecies distribution (Figs. S4–S6), the overall charge of the molecule, and the surface charge of the sorbent. A summary of interactions at pH ranges recommended for each sorbent is summarized in Fig. 3.

Oasis® HLB is a universal sorbent for acidic, neutral, and basic compounds. It is a hydrophilic-lipophilic-balanced, water-wettable, reversed-phase sorbent made from a specific ratio of two monomers, namely the hydrophilic *n*-vinylpyrrolidone and the lipophilic divinylbenzene [53]. The analytes can interact with this sorbent through dipole-dipole interactions and also through π-π interaction between the aromatic rings, as expected for PAG and EPS. TMAO, due to its small size and charged state (either positive or zwitterionic), cannot interact very efficiently with the neutral sorbent.

The carboxylic acid WCX (weak cation exchange sorbent, pK_a_ ~ 5) and the piperazine WAX (weak anion exchange, pK_a_ ~ 6–9) are derivatives of Oasis® HLB and provide both reversed-phase and ion-exchange retention mechanisms with the ability to retain and release strong bases and strong acids, respectively [53]. In the case of WCX, the sorbent surface is neutral at pH < 5, while all analytes are charged; thus, retention would not be satisfactory unless aromatic interactions are predominant in the case of EPS and PAG. At pH > 5, the sorbent is negatively charged while the analytes are also predominantly negatively charged, so electrostatic repulsion would not permit the retention of analytes. Only at pH 5–6 the retention of TMAO would be efficient thanks to the electrostatic interaction (sorbent negatively charged, and TMAO positively charged). For WAX, sorbent surface is positively charged at pH < 9, while TMAO is also positively charged, PAG is negatively charged approximately at pH between 3 and 9, and EPS is always negatively charged as a result of its low pK_a_. Therefore, only PAG and EPS would interact with the sorbent at pH 3–9. At pH > 9, the sorbent is neutral, and the analytes are mostly negatively charged; thus, retention would be, in the best case, due to π-π interactions.

The sulfonic acid MCX (mixed-mode cation exchange sorbent) and quaternary amine MAX (mixed-mode anion exchange) are also derivatives of Oasis® HLB and provide both reversed-phase and ion-exchange retention mechanisms, enabling greater cleanup selectivity and sensitivity for basic and acidic compounds, respectively [53]. In the case of MCX, which is permanently negative, under acidic conditions, TMAO and PAG are positively charged, allowing their retention. They can be easily eluted afterwards by increasing the pH and making them zwitterionic and negative, respectively. EPS cannot interact with this sorbent due to permanent electrostatic repulsion. For MAX, which is permanently positive, neutral to basic pH values permit the retention of EPS and PAG, which are negatively charged. TMAO would be neutral, so interactions would not be so efficient. PAG and TMAO can be eluted at low pH values, making them positive and thus favoring electrostatic repulsion between analytes and sorbent. This is not the case with EPS, which is always negative and therefore always interacts electrostatically with the sorbent, its desorption being hindered.

In summary, HLB can interact with the analytes thanks to both hydrophobic and hydrophilic interactions, being pH independent. WAX would predictably work for PAG and EPS, MCX for TMAO and PAG, and MAX for PAG.

As none of the sorbents is completely suitable for the extraction of all analytes, HLB and two different combinations of sorbents were tested: MCX + HLB (1:1, w/w) and MCX + WAX (1:1, w/w).

In order to assess the suitability of each sorbent/mixture, a mixed standard solution containing 200 ng mL^−1^ of each analyte was prepared in surrogate matrix and subjected to an adapted dispersive solid-phase extraction protocol, as described in Supplementary Information, prior to its injection in the LC-MS/MS system. The signal was compared to a solution of the same concentration prepared in the elution solvent to calculate EE. Three replicates of the extraction and analysis were performed for each tested sorbent.

**Fig. 4:**
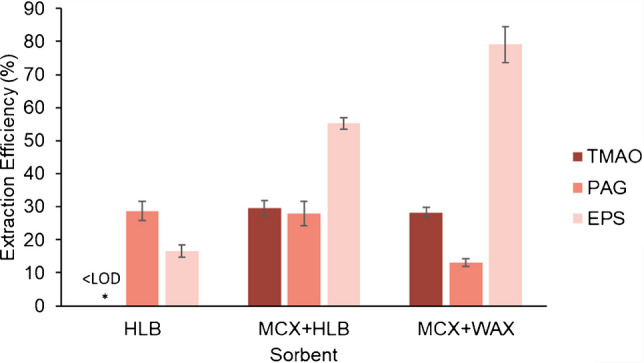
Extraction efficiency of different sorbent/mixtures. The bars represent the mean and the error bars represent the standard deviation of three replicates

Looking at Fig. 4, TMAO is not extracted by HLB, so the presence of MCX is necessary for this analyte. For PAG, HLB seemed to interact better than MCX and WAX. Comparing EPS results, in the presence of WAX (and, therefore, through an ion exchange mechanism) EEs are higher than in the presence of HLB. Even though MCX should not contribute to EPS extraction, it contributed to the effective desorption of the analyte in the elution step, increasing the negatively charged environment, thus favoring the transfer of the negative analyte to the elution solvent. In conclusion, the selected sorbent was the mixture of HLB and MCX because, on average, EEs were higher.

The sorbent was then magnetized as described in and characterized. The characterization of the material is described in Supplementary Information, including magnetization curve, morphology, adsorption-desorption isotherm, specific surface area, and pore size.

The performance of the magnetic sorbent was compared to bare MNPs by the extraction of a 200 ng mL^−1^ mixed standard solution, in which signals were below the LOD, showing that the polymer is responsible for the extraction.

Besides, three different batches of the magnetic sorbent were compared by extracting three replicates of this standard solution with each one of the three batches, achieving an RSD below 11% (for the nine replicates) and thus showing the good repeatability of the synthesis.

### Optimization of the mSBSDME extraction and desorption variables

A screening study was first performed to evaluate and select the important variables affecting the extraction and desorption performance. In this context, the Plackett-Burman design was used to simultaneously determine the influence of several factors on the analytical response (i.e., peak area) [54]. This design takes into account all variables affecting the mSBSDME procedure but assumes that interactions between them can be completely excluded. Therefore, the main effect is calculated by running a small number of experiments (e.g., 12 runs). The different variables (and their ranges) selected for this study were formic acid concentration in donor phase (0–800 mM, corresponding, approximately to pH 7–2, respectively), sorbent amount (1–5 mg), extraction time (1–10 min), desorption time (1–5 min), and NH_4_OH concentration in desorption solvent (0–0.1% (w/w)).

The Plackett-Burman design is shown in Table S2, and statistical analysis was performed using Minitab 19 (Minitab LLC, PA, USA) as the data processing software. All experiments were performed using 60 µL of surrogate matrix containing 200 ng mL^−1^ of target analytes and diluted up to 300 µL with an aqueous solution of formic acid to achieve the desired concentration in the donor phase. Desorption was performed using 30 µL of acetonitrile containing the necessary concentration of NH_4_OH.

**Fig. 5:**
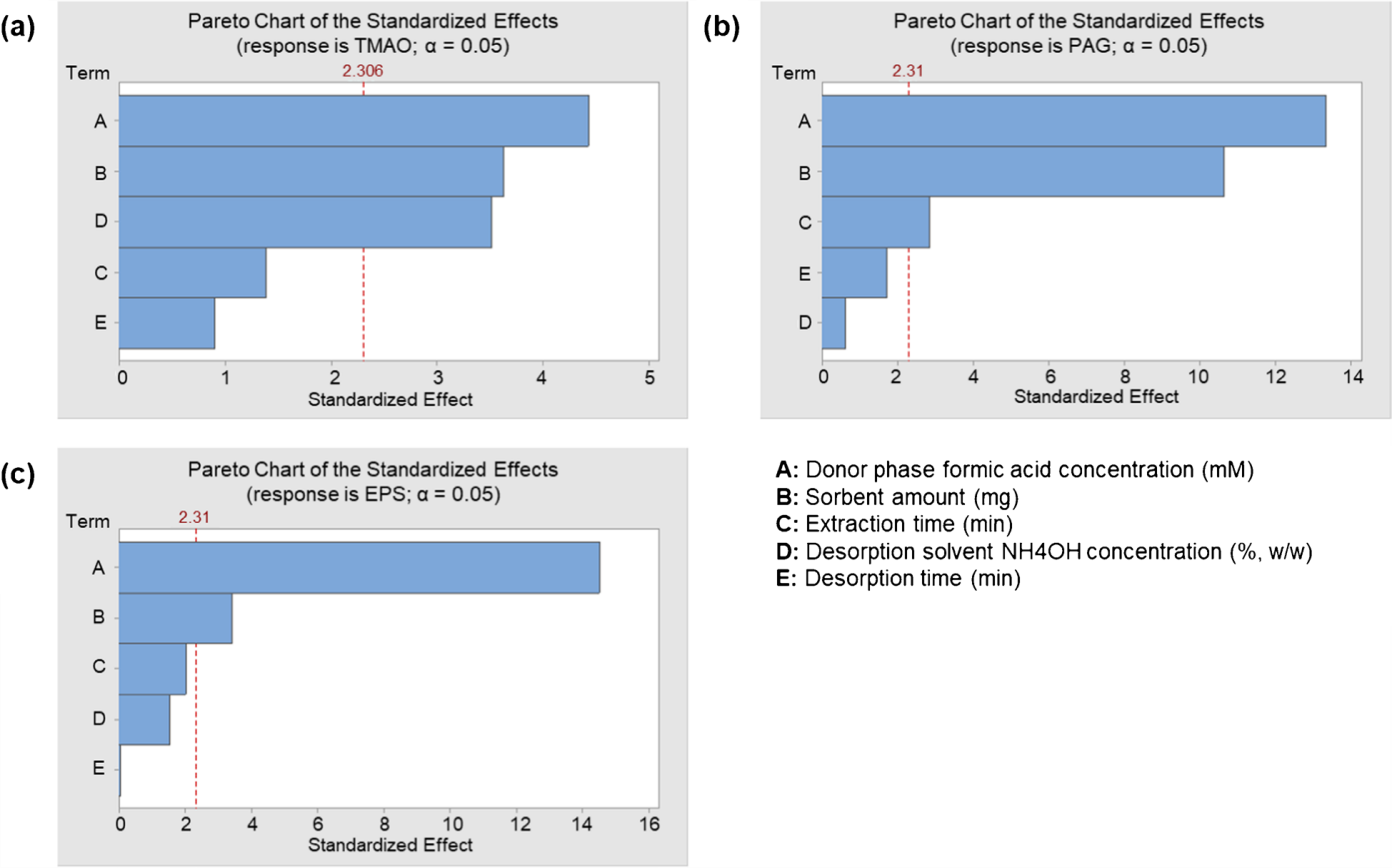
Pareto charts obtained in the Plackett-Burman design for **a** TMAO, **b** PAG, and **c** EPS

The results were evaluated using analysis of variance (ANOVA) and presented using Pareto diagrams. Under these conditions, satisfactory adjusted determination coefficients (82–97%) were obtained. Each Pareto chart, shown in Fig. 5, displays a vertical baseline corresponding to the 95% confidence interval, with the size of each bar representing the influence of the associated variable. Therefore, any variable that exceeds the vertical links can be considered statistically significant.

As can be seen, all the variables except desorption time had a significant effect on the mSBSDME procedure efficiency for, at least, one compound. Therefore, desorption time was fixed to the minimum value to achieve a faster method (i.e., 1 min).

Then, a response surface methodology (RSM) [55] based on a four-factor three-level Box-Behnken design was performed to establish the optimal values of the significant variables involved in the mSBSDME procedure. It was noticed that, in those experiments with high extraction times and high sorbent amounts from the Plackett-Burman design, the sorbent is highly solvated and cannot be completely retrieved by the magnet, and therefore it is lost in the washing step, leading to a lower signal in such experiments. Therefore, the maximum sorbent amount was established at 3 mg for the Box-Behnken design, even though the maximum extraction time was kept. Hence, the studied variables (and their ranges) were formic acid concentration in donor phase (0–800 mM), sorbent amount (1–3 mg), extraction time (1–10 min), and NH_4_OH concentration in desorption solvent (0–0.1% (w/w)). The same conditions used for Plackett-Burman were employed for this set of experiments, but setting the desorption time to 1 min.

The selected analytical responses were the peak area of each analyte. The description of the statistics of the Box-Behnken design is included in Supplementary Information. A total of 27 experiments including 3 replicates of the central point were performed (Table S3). StatGraphics Centurion XVI (Stat Point Inc. Herndon, VA, USA) software was employed for the statistical analysis.

**Fig. 6:**
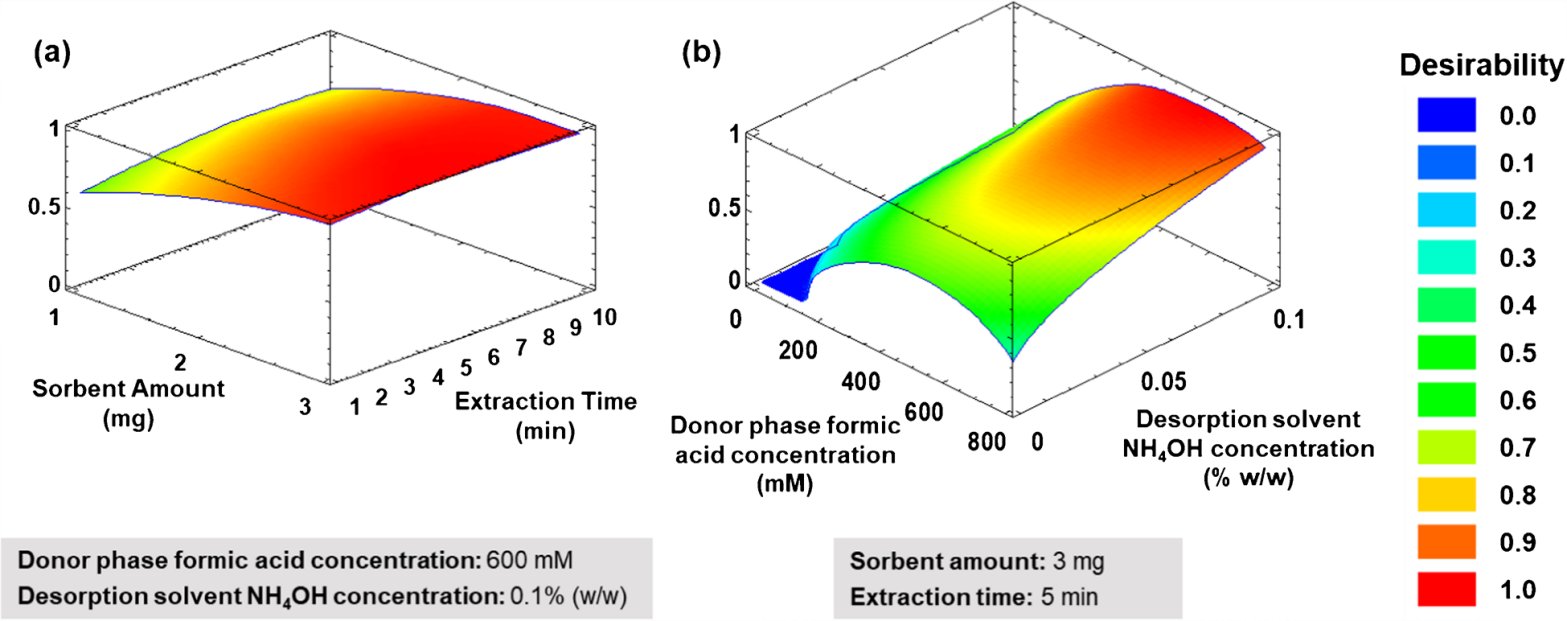
Response surface of the desirability function representing the relation between the studied variables: **a** sorbent amount vs extraction time, and **b** formic acid concentration in donor phase vs NH_4_OH concentration in desorption solvent

Under these conditions, satisfactory adjusted determination coefficients (84–98%) were obtained. Figure 6 shows the response surfaces of the desirability function. As can be seen, extraction performance improves at higher sorbent amounts, even though it starts to stabilize above 2.5 mg most probably due to the inability of the magnet to retrieve all the material. Therefore, 3 mg were selected. As expected from Plackett-Burman, extraction time was only significant for the extraction of PAG; therefore, for the overall effect, the extraction time was not very significant. However, a slight increase in the signal can be observed from 1 to 5 min; therefore, an extraction time of 5 min was selected.

Regarding the concentration of formic acid in the donor phase (i.e., pH), an acidic pH favors the interaction of the analytes with the sorbent. Nonetheless, at very low pH, the solvation of the material is enhanced, and the magnetic retrieval is hindered. Thus, a formic acid concentration of 600 mM (pH ~ 2) was selected.

For the desorption step, a highly basic media was needed to achieve an effective desorption, especially for TMAO, which is mainly retained by ionic interactions. Therefore, acetonitrile containing 0.1% (w/w) of NH_4_OH was selected as desorption solvent.

### Analytical performance of the proposed method

The results of method validation are summarized in Table 3.

**Table 3:** Analytical figures of merit of the proposed method

**Analyte**		**TMAO**	**PAG**	**EPS**
**Equation**		y = 0.0019x + 0.0232	y = 0.0021x + 0.0127	y = 0.0015x + 0.0111
**R**^**2**^		0.996	0.993	0.996
**EE (%)**^**a**^		7 ± 1	47 ± 3	48 ± 2
**LOD (ng mL**^**−1**^**)**^**b**^		22	2	9
**LOQ (ng mL**^**−1**^**)**^**b**^		74	6	30
**Accuracy (%)**^**c**^	**30 ng mL**^**−1**^	-^d^	91 ± 10	97 ± 8
	**400 ng mL**^**−1**^	98 ± 4	101 ± 3	101 ± 2
	**800 ng mL**^**−1**^	93 ± 4	101 ± 4	98 ± 4

^a^*EE*, extraction efficiency

^b^*LOD*, limit of detection; *LOQ*, limit of quantification; calculated as 3 and 10 times, respectively, the standard deviation of the signal of a blank (n = 9)

^c^Mean of five replicates ± standard deviation

^d^Concentration below LOQ

The results indicated that the linearity reached at least 1000 ng mL^−1^ for the target analytes. The determination coefficient revealed good linearity (R^2^ ≥ 0.993).

The method LOQs, in the low ng mL^−1^ level, allow the determination of the target analytes in human plasma at the expected levels [38–40], proving its suitability.

EEs of 7, 47, and 48 were achieved for TMAO, PAG, and EPS, respectively. Even though these values are not very high (especially for TMAO), the proposed method allows reaching very low LOQs at the ng mL^−1^ level, and the analytes are expected to be present at higher concentrations in plasma samples. Besides, the clean-up step is necessary due to the high saline and protein concentration of the plasma samples, which cannot be introduced directly into the MS instrument.

Accuracy results, presented in Table 3, are calculated as the ratio (in percentage) of the calculated and nominal concentrations of five replicates of the QCs. Mean and standard deviation values of these five replicates are presented in Table 3. Precision results, as RSD, are not explicitly reported in the table because the standard deviation of accuracy is presented instead. The values of accuracy around 100% and the standard deviations equal or below 10% revealed that good accuracy and precision were achieved for the target analytes in most cases.

### Analysis of clinical samples

Plasma samples obtained from participants in a clinical study, divided into low and high anxiety groups, were treated by the proposed mSBSDME approach and measured by LC-MS/MS. The obtained results are presented in Table 4.

**Table 4:** Measured concentration in human plasma samples

Group	Sample	Concentration (ng mL^−1^)		
		TMAO	PAG	EPS
Low anxiety	A	1580 ± 40	225 ± 2	118 ± 2
	B	558 ± 3	1130 ± 50	117 ± 7
	C	6620 ± 190	630 ± 20	228 ± 6
	D	173 ± 8	750 ± 30	59 ± 11
High anxiety	E	400 ± 20	576 ± 9	30.6 ± 0.5
	F	677 ± 11	295 ± 14	< LOQ
	G	1377 ± 16	549 ± 6	< LOQ
	H	314.0 ± 0.4	772 ± 12	106 ± 2

A *t*-test was applied to both groups of samples to determine if they were statistically different. A *p*-value > 0.05 indicated that both groups are not statistically different. A wider study with a larger cohort of patients is needed to conclude the clinical relevance of these analytes. At this point, it should be noted that the identity of EPS could not be confirmed because the ion ratios of the quantifier and qualifier ions in some cases were not within the acceptable range [57]. This is mainly due to the low abundance of the qualifier ion, whose variation at very low concentrations is higher than desirable.

In a subsequent experiment, a plasma sample was spiked at 150 ng mL^−1^ to evaluate the matrix effects by means of the relative recovery (RR, %) values, calculated as follows:

1 $$\%\;RR=\frac{{Concentration}_{spiked\;sample}-{Concentration}_{sample}}{Spiked\;amount}\cdot100$$

RR values were 88 ± 8%, 104 ± 5%, and 84 ± 8% for TMAO, PAG, and EPS, respectively. From these results, we can conclude that surrogate matrix calibration employing deuterated internal standards is effective to achieve negligible matrix effects.

Fig. S10 shows a chromatogram of this plasma sample before and after spiking.

### Comparison with other methods

To evaluate the performance of the proposed method, a comparison was made with a selection of previously reported analytical methods for the determination of TMAO, PAG, and EPS in serum/plasma samples (Table 5). The analytical methods for this comparison were selected from those published in the last 5 years or if they allowed the determination of more than one of the target compounds.

In comparison with previously reported methods, the proposed method demonstrates competitive sensitivity, achieving a LOD for PAG and EPS among the lowest reported. While the method involves additional steps due to the mSBSDME procedure, this approach provides a novel alternative for plasma samples, offering satisfactory recovery and precision. Besides, sample consumption is relatively low and sample preparation and analysis times are short. Furthermore, the proposed method is the only one capable of simultaneously analyzing the three target analytes in a single run thanks to the combination of sorbent materials, making it particularly valuable for future comprehensive studies involving these compounds.

**Table 5:** Comparison of the proposed method with other approaches for the determination of TMAO, PAG and/or EPS in plasma or serum samples

Analytes^a^	Sample	Sample preparation	Technique	Sample preparation time (min/sample)^b^	Analysis time (min)^c^	LOD (ng mL^−1^)^d^	RSD (%)^e^	RR (%)^f^	Ref
TMAO	Plasma (50 µL)	Derivatization	LC-MS	2.0	16	4.1	8.5	86–95	[18]
TMAO, PAG	Serum (25 µL)	Protein precipitation	LC-MS/MS	5.9	14	10 (TMAO); 5 (PAG)	< 6.3	90–104	[19]
TMAO	Plasma (50 µL)	Protein precipitation + Derivatization	LC-MS/MS	1.3	5	n.r.^**g**^	< 5.8	n.r.^**g**^	[21]
TMAO, EPS	Plasma/ Serum (10 µL)	Protein precipitation + Evaporation/reconstitution	LC-MS/MS	4.3	20	30.0 (TMAO); 20.2 (EPS)	< 10.7	92–106	[22]
TMAO	Serum (0.2 g)	Protein precipitation	2D-LC-MS/MS	3.6	14	10.3	< 3.5	96–108	[23]
TMAO, PAG	Serum (200 µL)	Ultracentrifugation/Protein precipitation	LC-MS/MS	11.7	25	n.r.^**g**^	< 12	n.r.^**g**^	[36]
PAG, EPS	Serum (25 µL)	Ultracentrifugation/Protein precipitation	LC-MS/MS	1.8	6	n.r.^**g**^	n.r.^**g**^	n.r.^**g**^	[37]
EPS	Serum (100 µL)	Protein precipitation	LC-MS/MS	1.8	15	16.9	< 6	71–118	[38]
TMAO, PAG, EPS	Plasma (60 µL)	mSBSDME	LC-MS/MS	2.3	4	22 (TMAO); 2 (PAG); 9 (EPS)	< 10	84–104	This work

^a^Only the analytes involved in this work are reported even though the works may determine other compounds

^b^Estimated considering all the sample preparation steps in multiple position instruments, when applicable, based on [57]

^c^Total run time of chromatogram

^d^*LOD*, limit of detection

^e^*RSD*, relative standard deviation

^f^*RR*, relative recoveries

^g^*n.r.*, not reported

## Conclusions

This work describes a method based on mSBSDME followed by LC-MS/MS analysis for the determination of three gut microbiome metabolites (TMAO, PAG, EPS) in human plasma samples. A magnetic composite made of CoFe_2_O_4_ MNPs embedded into a mixture of commercial sorbents (hydrophilic-lipophilic balance and mixed-mode cation exchange) allowed the simultaneous extraction of the three analytes. After the thoughtful study of the chromatographic conditions, and the optimization of extraction and desorption steps, this approach demonstrated competitive sensitivity, with LODs of a few nanograms per milliliter, and satisfactory accuracy and precision. Notably, it is the first reported method capable of analyzing these three compounds simultaneously, addressing a significant gap in the field for comprehensive studies with clinical application. Some issues still need to be addressed, such as improving sensitivity for TMAO and ensuring identification of EPS and, therefore, they constitute the focus of future improvements of the method.

## Supplementary Information

Below is the link to the electronic supplementary material.

Supplementary file1 (PDF 1.16 MB)

## Data Availability

The data that support the findings of this study are available on request from the corresponding author.
