# Supplementary material for: Mixed sorbent in miniaturized stir bar sorptive dispersive microextraction for the determination of gut microbiome metabolites in plasma samples
Source: Anal Bioanal Chem. 2026 Feb 19;418(9):2641–55. doi: 10.1007/s00216-026-06391-8 (PMC13079491; doi:10.1007/s00216-026-06391-8)
Supplement: Supplementary file 1 — Supplementary file1 (PDF 1.16 MB) [file 216_2026_6391_MOESM1_ESM.pdf]

Supplementary Information

Mixed sorbent in miniaturized stir bar sorptive dispersive microextraction for the determination of gut microbiome metabolites in plasma samples

Cristian Azorín[a,b,*], Sara R. Fernandes[b,c], Luisa Barreiros[b,c], Juan L. Benedé[a], Alberto Chisvert[a], Marcela A. Segundo[b]

[a] GICAPC Research group, Department of Analytical Chemistry, University of Valencia, 46100 Burjassot, Valencia, Spain

[b] LAQV, REQUIMTE, Department of Chemical Sciences, Faculty of Pharmacy, University of Porto, Rua de Jorge Viterbo Ferreira 228, 4050-313, Porto, Portugal

[c] ESS, Polytechnic of Porto, Rua Dr. António Bernardino de Almeida 400, 4200-072 Porto, Portugal

* Corresponding author:

e-mail address: cristian.azorin@uv.es

Table of contents

Preparation of synthetic plasma 2
MS/MS parameters 2
Product ion spectra 3
Microspecies distribution *vs* pH 6
Dispersive solid phase extraction for sorbent selection 8
Characterization of sorbent material 9
  Instruments for composite characterization 9
  Magnetization curve 9
  Morphology 10
  Adsorption-desorption isotherm, specific surface area and pore size 10
Plackett-Burman design 12
Box-Behnken design 13
Chromatograms of samples 15
References 16

Preparation of synthetic plasma

Synthetic plasma employed in the optimization and validation of the method was prepared according to an adapted protocol from UNE-EN ISO 10993-15:2009 [1]. For that aim, an aqueous solution containing 6.8 g L^{-1} NaCl, 0.2 g L^{-1} $CaCl_2$, 0.4 g L^{-1} KCl, 0.1 g L^{-1} $MgSO_4$, 2.2 g L^{-1} $NaHCO_3$, 0.16 g L^{-1} $Na_2HPO_4{\cdot}2H_2O$, and 0.02 g L^{-1} NaH_2PO_4 in ultrapure water was prepared, adjusted to pH 7.4 with HCl 0.1 M and kept at 4 °C. Each day before using, 60 g L^{-1} of bovine serum albumin (BSA) were added to the required volume of the previous solution to simulate protein content of plasma.

MS/MS parameters

Table S1. MRM data for identification and quantification (in bold) of the target compounds.

Analyte	Polarity	Precursor ion (*m/z*)	Product ion (*m/z*)	Collision energy (V) [a]
TMAO	+	**76.2**	**58.2**	**-23.0**
			42.2	-40.0
PAG	+	**265.2**	**130.1**	**-16.0**
			91.2	-42.0
EPS	-	**200.9**	**121.2**	**19.0**
			80.0	40.0
TMAO-d_9	+	**85.2**	**66.2**	**-25.0**
			46.3	-44.0
PAG-d_5	+	**269.8**	**130.1**	**-18.0**
			96.3	-17.0
EPS-d_4	-	**204.9**	**125.1**	**23.0**
			80.0	15.0

[a] Expressed as voltage as set in LabSolutions software.

Product ion spectra

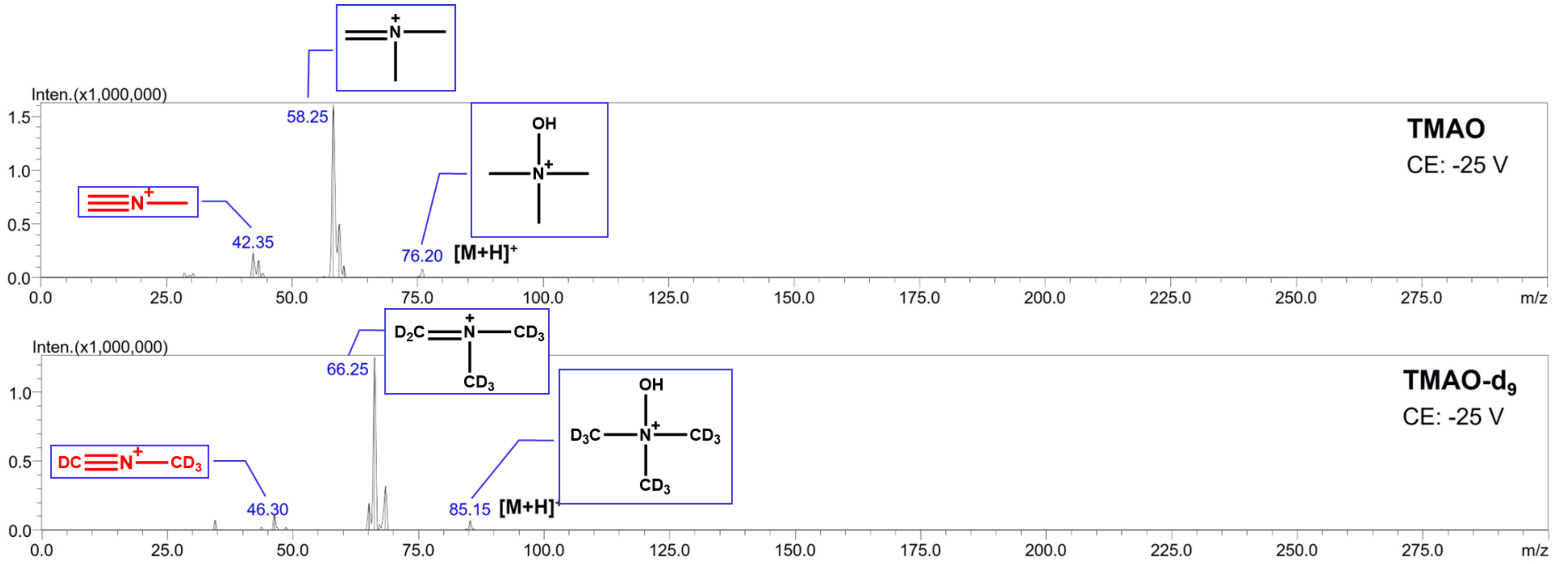

Fig. S1. MS/MS product ion spectra for precursor ion $[M+H]^+$ of TMAO and TMAO-d_9 at *m/z* 76.20 and 85.15, respectively, obtained at a collision energy value of -25.0 V. Black structures represent pseudomolecular ions and previously reported product ions [2], while red structures represent proposed structures for product ions predicted by CFM-ID 4.0 spectrum prediction tool [3].

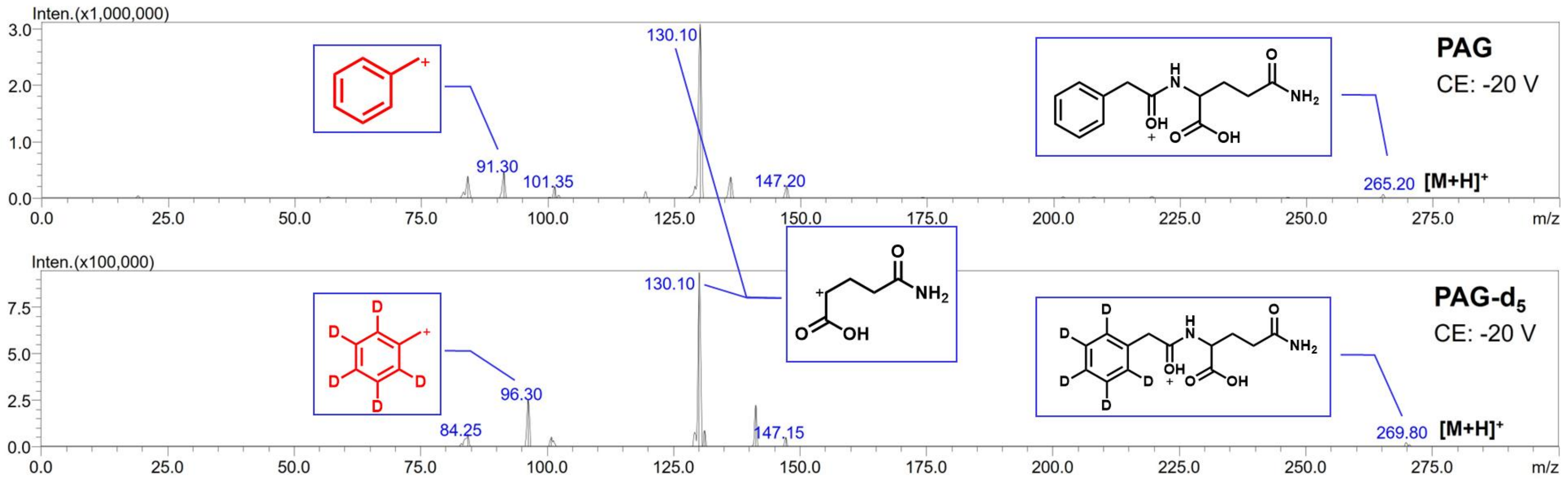

Fig. S2. MS/MS product ion spectra for precursor ion [M+H]$^+$ of PAG and PAG-d$_5$ at *m/z* 265.20 and 269.80, respectively, obtained at a collision energy value of -20.0 V. Black structures represent pseudomolecular ions and previously reported product ions [4], while red structures represent proposed structures for product ions predicted by CFM-ID 4.0 spectrum prediction tool [3].

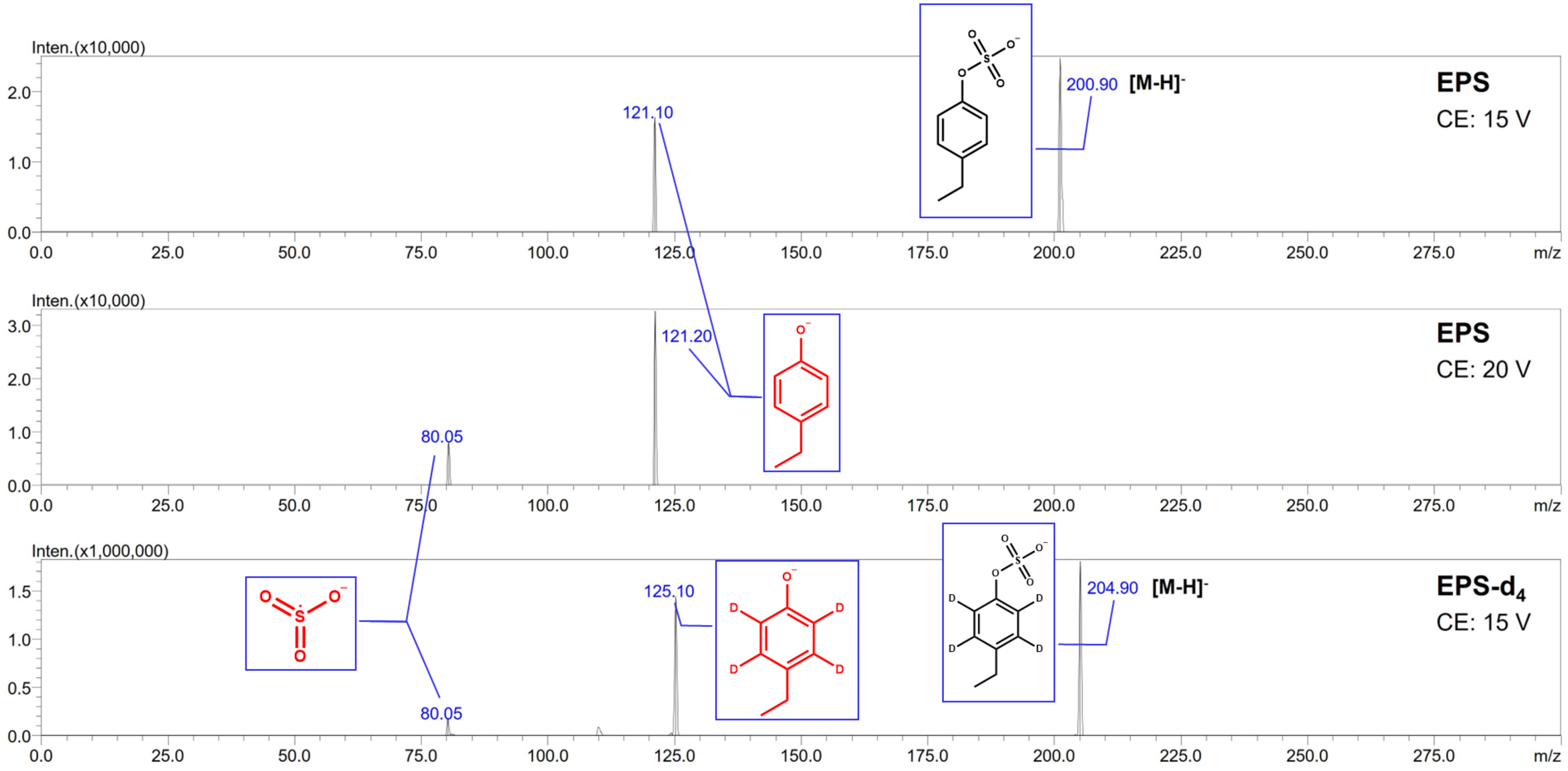

Fig. S3. MS/MS product ion spectra for precursor ion $[M-H]^-$ of EPS and EPS-d_4 at *m/z* 200.90 and 204.90, respectively, obtained at a collision energy value of 15.0 and 20.0 V. Black structures represent pseudomolecular ions, while red structures represent proposed structures for product ions predicted by CFM-ID 4.0 spectrum prediction tool [3], which is consistent with previously reported fragmentation mechanisms of sulfate metabolites [5].

Microspecies distribution *vs* pH

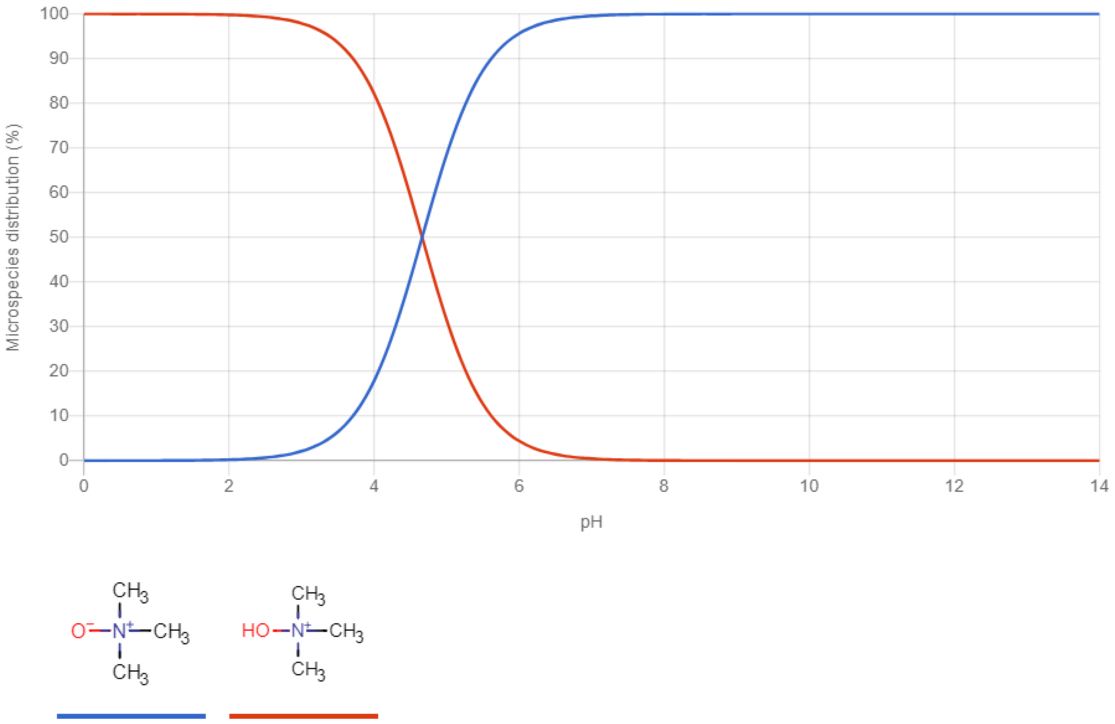

Fig. S4. Trimethylamine *N*-oxide structure (TMAO) featuring group ionization at different pH values. Calculations were performed using Chemicalize software (https://chemicalize.com).

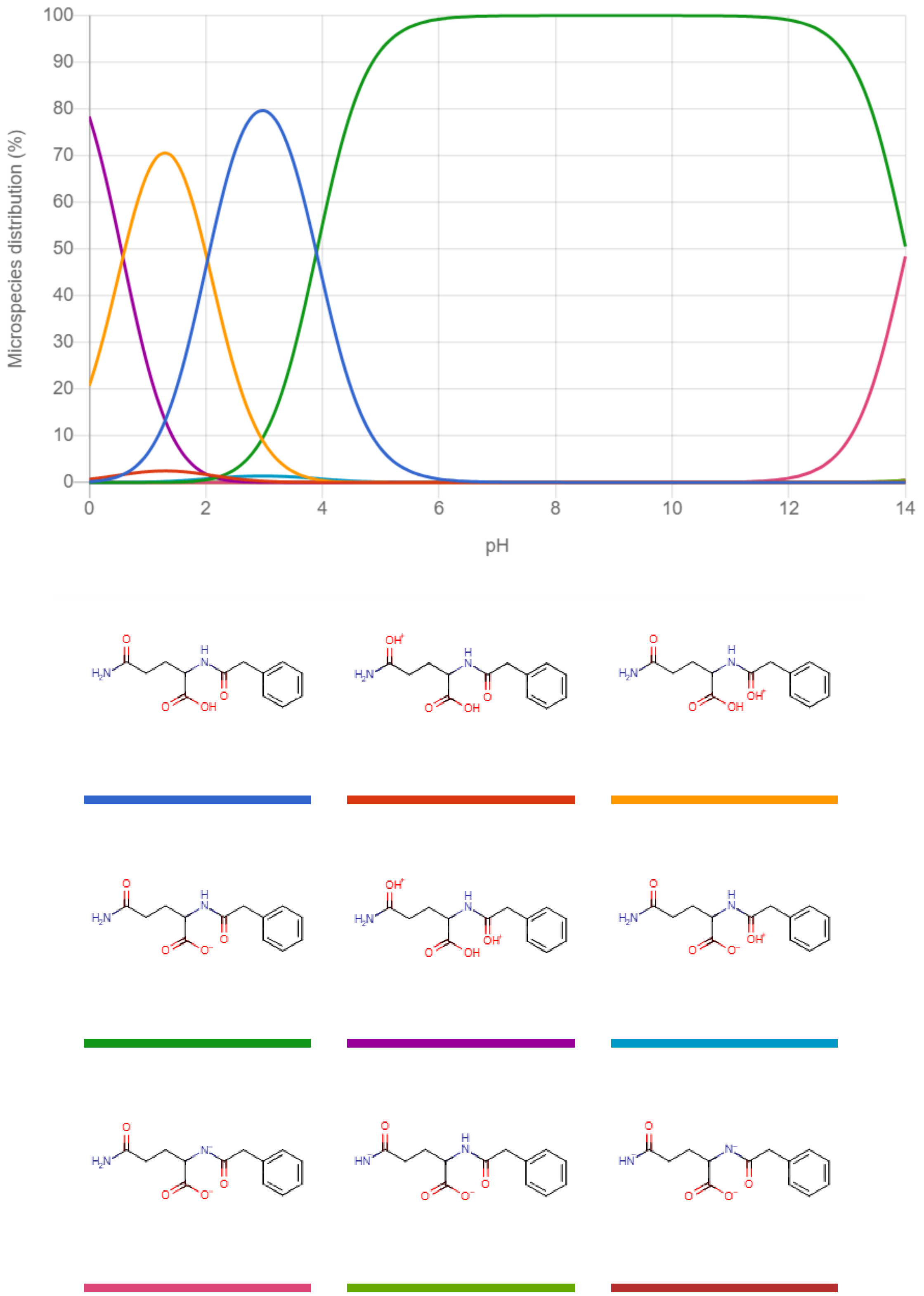

Fig. S5. Phenylacetylglutamine (PAG) structure featuring group ionization at different pH values. Calculations were performed using Chemicalize software (https://chemicalize.com).

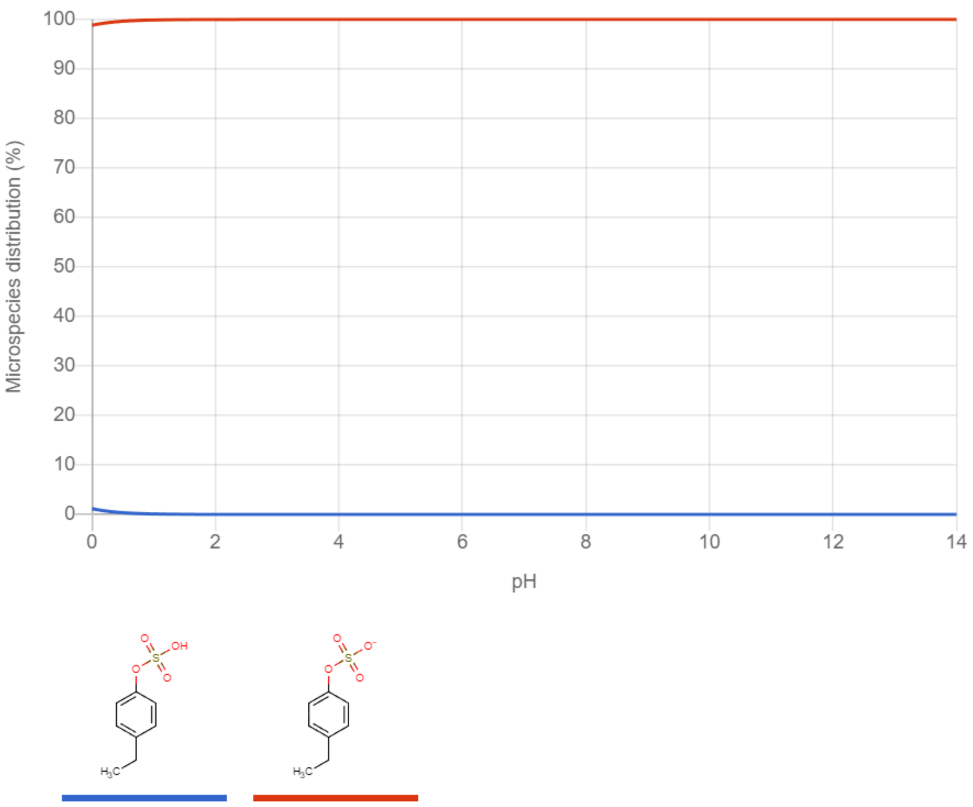

Fig. S6. 4-Ethylphenyl sulfate (EPS) structure featuring group ionization at different pH values. Calculations were performed using Chemicalize software (https://chemicalize.com).

Dispersive solid phase extraction for sorbent selection

Protocols were adapted from generic SPE methods described by the supplier [6].

- Protocol for Oasis HLB:

  0.5 mL of standard solution prepared in synthetic plasma were diluted up to 1 mL with ultra-pure water, maintaining pH ~ 7.4. 30 mg of sorbent were added and vortex-mixed for 1 min. Then it was centrifuged at 13400 rpm for 2 min and the supernatant was discarded. For washing, 1 mL of ultra-pure water was added, vortexed for 1 min and separated again by centrifugation. Finally, elution was carried out by adding 1 mL of methanol, which was vortexed for 1 min and separated by centrifugation. The supernatant was transferred to an injection vial for its analysis.

- Protocol for mixtures MCX+HLB and MCX+WAX:

  0.5 mL of standard solution prepared in synthetic plasma were diluted up to 1 mL with 20% (v/v) formic acid, achieving a pH ~ 2. 30 mg of sorbent were added and vortex-mixed for 1 min. Then it was centrifuged at 13400 rpm for 2 min and the supernatant was discarded. For washing, 1 mL of 2% (v/v) formic acid was added, vortexed for 1 min and separated again by centrifugation. Finally, elution was carried out by adding 1 mL of methanol containing 0.1% (w/w) of NH_4OH, which was vortexed for 1 min and separated by centrifugation. The supernatant was transferred to an injection vial for its analysis.

Characterization of sorbent material

Instruments for composite characterization

Different techniques were applied to characterize the $CoFe_2O_4$-MCX+HLB composite.

A Quantum Design (San Diego, CA, USA) MPMS-XL-5 superconducting quantum interference device (SQUID) magnetometer was used to measure the magnetic properties of the composite material.

A HITACHI (Tokyo, Japan) S-4800 scanning electron microscope (SEM) operating at 10 kV equipped with an RX Bruker (Billerica, MA, USA) backscattered electron detector, was used to observe the morphology and to perform energy-dispersive X-ray spectroscopy (EDS) of the composite.

Nitrogen adsorption-desorption isotherms were measured on an ASAP 2010 analyzer from Micromeritics (Norcross, GA, USA), in order to determine the surface area and pore size.

Magnetization curve

The magnetization curve (Fig. S4) showed a saturation magnetization (M_S) of 32.3 emu g^{-1}, which was appropriate to satisfactorily perform the mSBSDME procedure. A residual magnetism (B_r) of 15.8 emu g^{-1}, and a coercivity of about 1.7 kOe to demagnetize the sorbent after its magnetization were obtained. According to these results, the synthesized $CoFe_2O_4$-MCX+HLB composite is a soft ferromagnetic material.

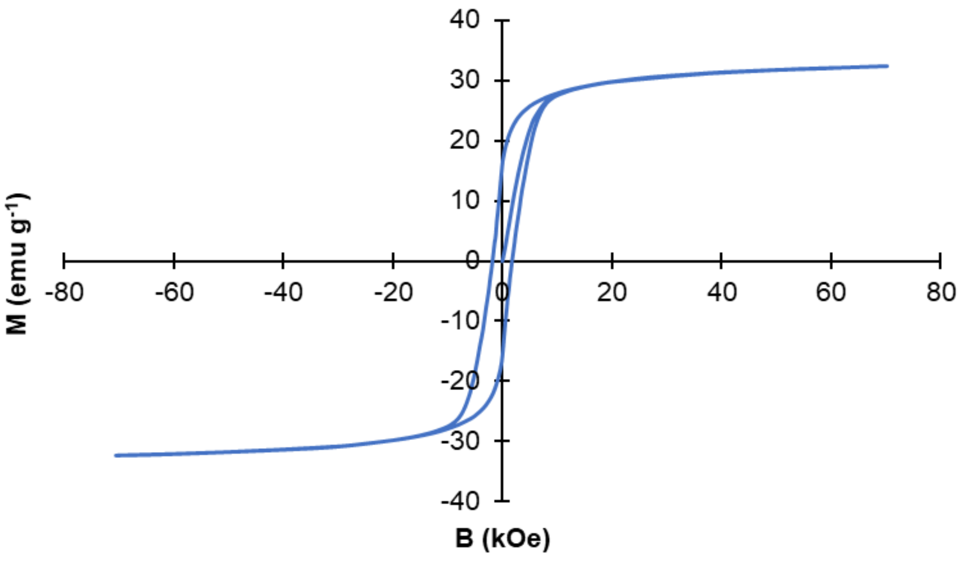

Fig. S7. Magnetization curve of the $CoFe_2O_4$-MCX+HLB composite.

Morphology

Morphology of the $CoFe_2O_4$-MCX+HLB composite was studied by SEM operating at 10 kV. Results are shown in Fig. S5. Specifically, a) and b) for the individual materials (*i.e.,* $CoFe_2O_4$ MNPs and Oasis MCX + Oasis HLB, respectively), and c) and d) for the resulting composite at different magnification levels. In these latter two images it can also be observed how the commercial beads are covered by the MNPs, resulting in the final magnetic sorbent material.

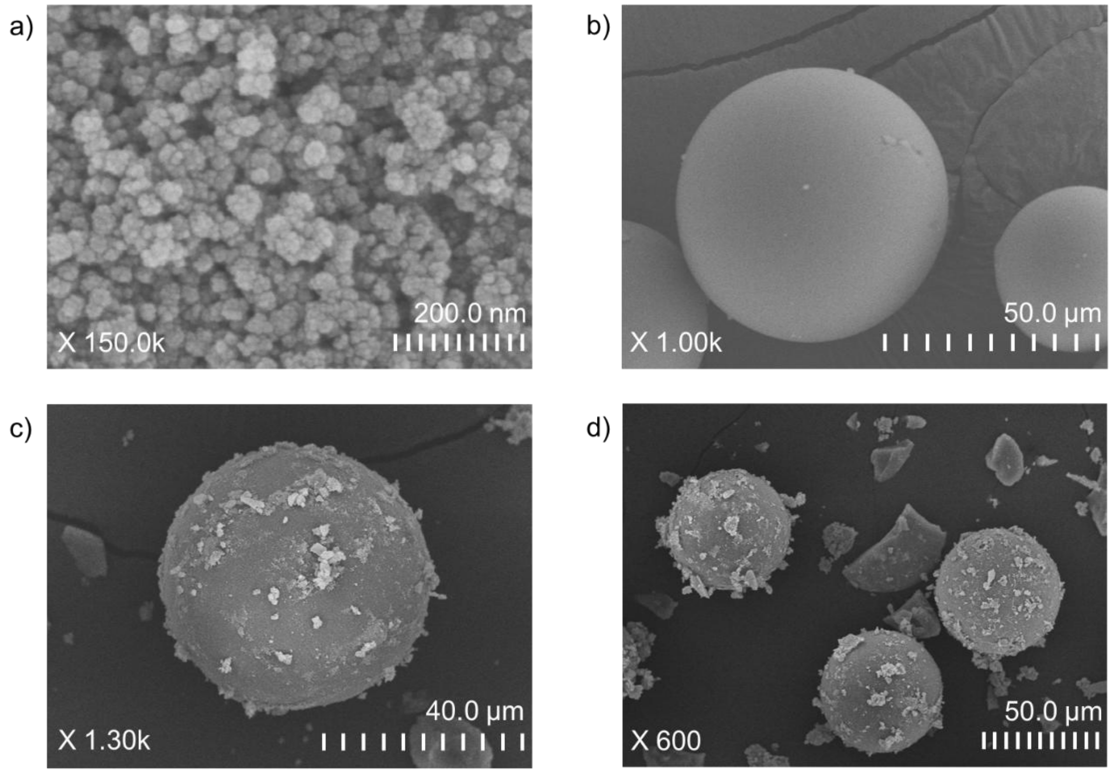

Fig. S8. (a) SEM micrograph of $CoFe_2O_4$ MNPs at magnification 150000; (b) SEM micrograph of a mixture of Oasis MCX and Oasis HLB at magnification 1000; (c) and (d) SEM micrographs of $CoFe_2O_4$-MCX+HLB composite at magnification 1300 (c) 600 (d).

Adsorption-desorption isotherm, specific surface area and pore size

The adsorption-desorption isotherm, along with the specific surface area and porosity, were measured to analyze the adsorption properties. According to the International Union of Pure and Applied Chemistry (IUPAC), most isotherms can be categorized into six types (I-VI) based on the interactions between the adsorbate and adsorbent, as well as between adsorbate molecules. Additionally, various shapes of hysteresis loops can occur due to the different pore structures of materials [7, 8]. In this study, the obtained adsorption-desorption isotherm is classified as a type-IV isotherm (See Fig. S6), with the hysteresis loop corresponding to a type H1 loop [7, 8]. Type-IV isotherm indicates mesoporosity and this hysteresis loop is typically associated with uniform mesopores. The average pore size was found to be 7.2 nm and the Brunauer-Emmett-Teller (BET) surface area was measured to be 378.9 ± 0.8 $m^2\ g^{-1}$.

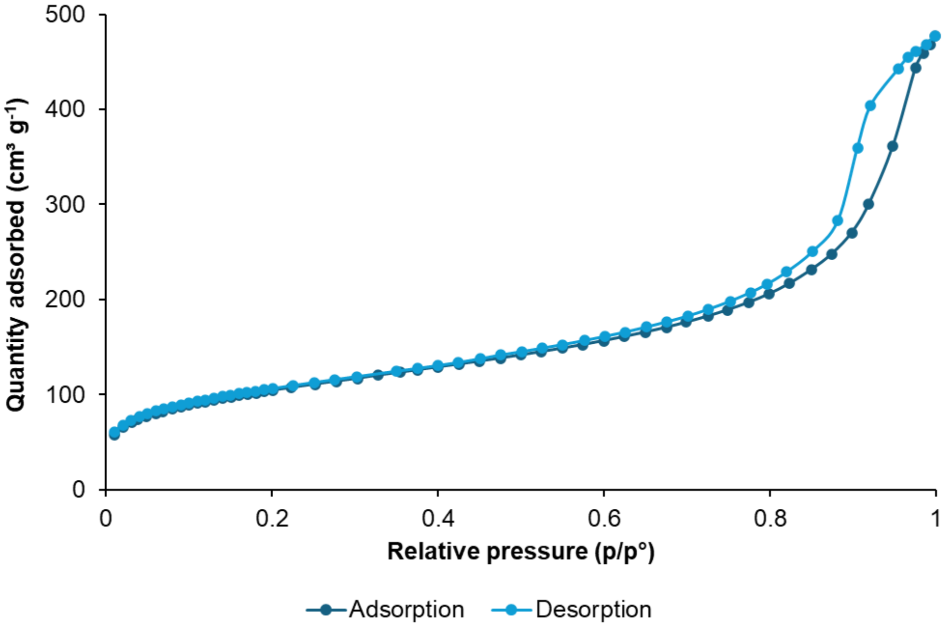

Fig. S9. Isotherm linear plot of the $CoFe_2O_4$-MCX+HLB composite.

Plackett-Burman design

Table S2. Plackett-Burman design for the screening of the extraction and desorption variables.

Run	Donor phase formic acid concentration (mM)		Sorbent amount (mg)		Extraction time (min)		Desorption solvent NH_4OH concentration (%, w/w)		Desorption time (min)	
	Uncoded	Coded	Uncoded	Coded	Uncoded	Coded	Uncoded	Coded	Uncoded	Coded
1	800	1	1	-1	10	1	0	-1	1	-1
2	800	1	5	1	1	-1	0.1	1	1	-1
3	0	-1	5	1	10	1	0	-1	5	1
4	800	1	1	-1	10	1	0.1	1	1	-1
5	800	1	5	1	1	-1	0.1	1	5	1
6	800	1	5	1	10	1	0	-1	5	1
7	0	-1	5	1	10	1	0.1	1	1	-1
8	0	-1	1	-1	10	1	0.1	1	5	1
9	0	-1	1	-1	1	-1	0.1	1	5	1
10	800	1	1	-1	1	-1	0	-1	5	1
11	0	-1	5	1	1	-1	0	-1	1	-1
12	0	-1	1	-1	1	-1	0	-1	1	-1
13	400	0	3	0	5.5	0	0.05	0	3	0
14	400	0	3	0	5.5	0	0.05	0	3	0
15	400	0	3	0	5.5	0	0.05	0	3	0

Box-Behnken design

The Box-Behnken designs are a class of rotatable or nearly rotatable second-order designs based on three-level incomplete factorial designs. The number of experiments required for the development of the designs (N) is defined as follows:

$$N=2k(k-1)+C_P \quad (1)$$

where k is the number of factors (*i.e.*, 4); and C_P is the replicates of the central point. In this sense, performing 3 replicates of the central point and according to Eq. 1, 27 experiments were required. The range of each factor (Table S3) was defined between a high and a low value.

The following quadratic polynomial equation was applied to evaluate the multiple linear regression for each response:

$$Y=\beta_o+\sum \beta_i X_i+\sum \beta_{ii} X_i^2+\sum \beta_{ij} X_i X_j+\varepsilon \quad (2)$$

where Y is the analytical response; β_o is a constant term; β_i, β_j, and β_{ij} are the regression coefficients of the design; X_i and X_j are the different variables; and ε is the residual error.

The global desirability of the experiment (D) was applied to evaluate the different responses and to achieve the optimal extraction conditions to maximize the analytical responses of the analytes:

$$D=\left(d_1(Y_1)\cdot d_2(Y_2)\cdots d_n(Y_n)\right)^{1/n} \quad (3)$$

where n is the number of responses in the optimization process, and $d_i(Y_i)$ is the individual desirability of each response in the experiment. The individual desirability is calculated as:

$$d_i=\frac{Y_i-Y_{min}}{Y_{max}-Y_{min}} \quad (4)$$

Undesirable responses and fully desirable responses are ranged between 0 and 1, respectively, for each response.

Table S3. Box-Behnken design for multivariate optimization of the critical variables.

Step	Donor phase formic acid concentration (mM)		Sorbent amount (mg)		Extraction time (min)		Desorption solvent NH_4OH concentration (%, w/w)	
	Uncoded	Coded	Uncoded	Coded	Uncoded	Coded	Uncoded	Coded
1	400	0	1	-1	1	-1	0.05	0
2	400	0	3	1	1	-1	0.05	0
3	400	0	1	-1	10	1	0.05	0
4	400	0	3	1	10	1	0.05	0
5	0	-1	2	0	5.5	0	0	-1
6	800	1	2	0	5.5	0	0	-1
7	0	-1	2	0	5.5	0	0.1	1
8	800	1	2	0	5.5	0	0.1	1
9	400	0	1	-1	5.5	0	0	-1
10	400	0	3	1	5.5	0	0	-1
11	400	0	1	-1	5.5	0	0.1	1
12	400	0	3	1	5.5	0	0.1	1
13	0	-1	2	0	1	-1	0.05	0
14	0	-1	2	0	10	1	0.05	0
15	800	1	2	0	1	-1	0.05	0
16	800	1	2	0	10	1	0.05	0
17	0	-1	1	-1	5.5	0	0.05	0
18	0	-1	3	1	5.5	0	0.05	0
19	800	1	1	-1	5.5	0	0.05	0
20	800	1	3	1	5.5	0	0.05	0
21	400	0	2	0	1	-1	0	-1
22	400	0	2	0	10	1	0	-1
23	400	0	2	0	1	-1	0.1	1
24	400	0	2	0	10	1	0.1	1
25	400	0	2	0	5.5	0	0.05	0
26	400	0	2	0	5.5	0	0.05	0
27	400	0	2	0	5.5	0	0.05	0

<u>**Chromatograms of samples**</u>

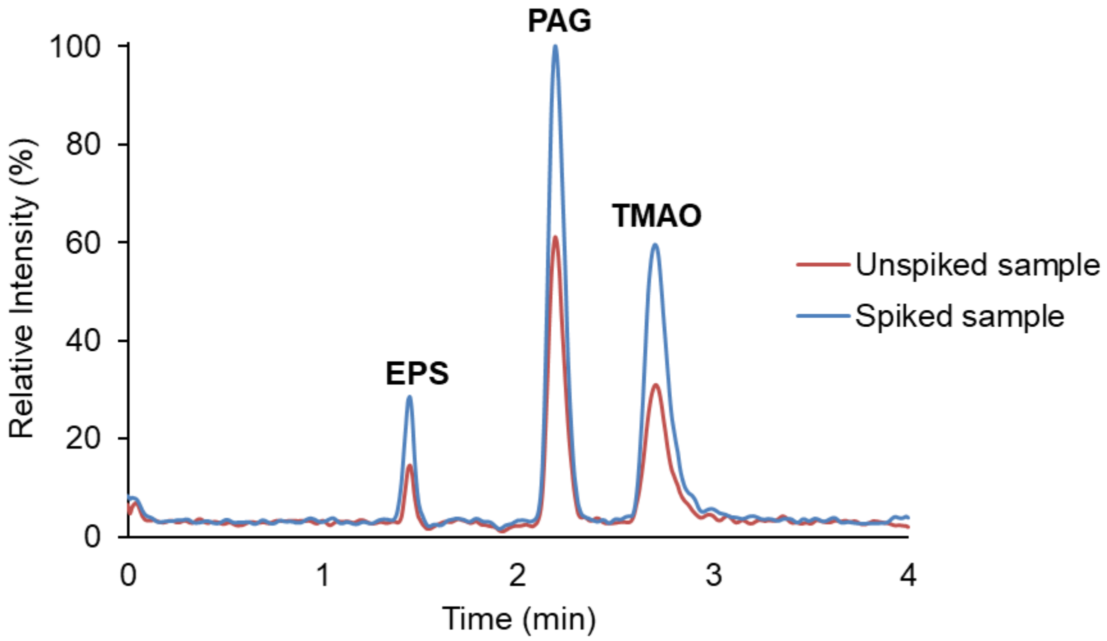

Fig. S10. Chromatogram obtained for a plasma sample before and after spiking with 150 ng mL^{-1} of the target analytes. The represented signal is the sum of the three quantitative transitions.

References

1. Kij A, Mateuszuk L, Sitek B, Przyborowski K, Zakrzewska A, Wandzel K, Walczak M, Chlopicki S (2016) Simultaneous quantification of PGI 2 and TXA 2 metabolites in plasma and urine in NO-deficient mice by a novel UHPLC/MS/MS method. J Pharm Biomed Anal 129:148–154. https://doi.org/10.1016/j.jpba.2016.06.050

2. Wang Z, Levison BS, Hazen JE, Donahue L, Li X-M, Hazen SL (2014) Measurement of trimethylamine-N-oxide by stable isotope dilution liquid chromatography tandem mass spectrometry. Anal Biochem 455:35–40. https://doi.org/10.1016/j.ab.2014.03.016

3. Wang F, Allen D, Tian S, Oler E, Gautam V, Greiner R, Metz TO, Wishart DS (2022) CFM-ID 4.0 – a web server for accurate MS-based metabolite identification. Nucleic Acids Res 50:W165–W174. https://doi.org/10.1093/nar/gkac383

4. Wiener EA, LeFevre GH (2022) White Rot Fungi Produce Novel Tire Wear Compound Metabolites and Reveal Underappreciated Amino Acid Conjugation Pathways. Environ Sci Technol Lett 9:391–399. https://doi.org/10.1021/acs.estlett.2c00114

5. Fernandes SR, Azorín C, Silva EMP, Miró M, Barreiros L, Segundo MA (2025) Targeted mass spectrometry method for the determination of multiple gut-microbiota metabolites in human plasma. J Chromatogr B 1265:124764. https://doi.org/10.1016/j.jchromb.2025.124764

6. Waters corporation (2017) Oasis HLB Cartridges and 96-well plates: Care and use manual. https://help.waters.com/content/dam/waters/ko/support/usermanuals/2020/715001391/715001391en.pdf. Accessed 20 Oct 2025

7. Sing KSW (1985) Reporting physisorption data for gas/solid systems with special reference to the determination of surface area and porosity (Recommendations 1984). Pure Appl Chem 57:603–619. https://doi.org/10.1351/pac198557040603

8. Thommes M, Kaneko K, Neimark A V., Olivier JP, Rodriguez-Reinoso F, Rouquerol J, Sing KSW (2015) Physisorption of gases, with special reference to the evaluation of surface area and pore size distribution (IUPAC Technical Report). Pure Appl Chem 87:1051–1069. https://doi.org/10.1515/pac-2014-1117
